# Fiber Optic-Based Durability Monitoring in Smart Concrete: A State-of-Art Review

**DOI:** 10.3390/s23187810

**Published:** 2023-09-11

**Authors:** Hou Qiao, Zhen Lin, Xiangtao Sun, Wei Li, Yangping Zhao, Chuanrui Guo

**Affiliations:** 1 College of Civil and Transportation Engineering, Institute of Urban Smart Transportation & Safety Maintenance, Shenzhen University, Shenzhen 518060, China; qiao_h@hdec.com (H.Q.); yangping@szu.edu.cn (Y.Z.); 2 Power China Huadong Engineering Corporation (HDEC), Hangzhou 311122, China; li_w@hdec.com; 3 Key Laboratory of Far-Shore Wind Power Technology of Zhejiang Province, Hangzhou 311122, China; 4 Department of Civil and Environmental Engineering, The Hong Kong Polytechnic University, Hung Hom, Kowloon, Hong Kong, China; zhenzz.lin@connect.polyu.hk; 5 Department of Disaster Mitigation for Structures, Tongji University, Shanghai 200092, China; sunxt@tongji.edu.cn

**Keywords:** durability monitoring, distributed fiber optic sensor, fiber Bragg grating, fluorescence effect, long-period fiber grating, surface plasma resonance, smart concrete

## Abstract

Concrete is the most commonly used construction material nowadays. With emerging cutting-edge technologies such as nanomaterials (graphene, carbon nanotubes, etc.), advanced sensing (fiber optics, computer tomography, etc.), and artificial intelligence, concrete can now achieve self-sensing, self-healing, and ultrahigh performance. The concept and functions of smart concrete have thus been partially realized. However, due to the wider application location (coastal areas, cold regions, offshore, and deep ocean scenarios) and changing climate (temperature increase, more CO_2_ emissions, higher moisture, etc.), durability monitoring (pH, ion penetration, carbonation, corrosion, etc.) becomes an essential component for smart concrete. Fiber optic sensors (FOS) have been widely explored in recent years for concrete durability monitoring due to their advantages of high sensitivity, immunity to harsh environments, small size, and superior sensitivity. The purpose of this review is to summarize FOS development and its application in concrete durability monitoring in recent years. The objectives of this study are to (1) introduce the working principle of FOS, including fiber Bragg grating (FBG), long-period fiber grating (LPFG), surface plasmon resonance (SPR), fluorescence-based sensors, and distributed fiber optic sensors (DFOS); (2) compare the sensitivity, resolution, and application scenarios of each sensor; and (3) discuss the advantages and disadvantages of FOS in concrete durability monitoring. This review is expected to promote technical development and provide potential research paths in the future for FOS in durability monitoring in smart concrete.

## 1. Introduction

Concrete is the most consumed construction material in the world. A total of 4.1 billion metric tons of cement were utilized to build concrete structures including buildings, bridges, and tunnels every year from 2018 to 2020 [1]. The deterioration of concrete may occur due to harsh environments, heavy service loads, initial micro-cracks, and enduring chemical reactions, including the alkali–silica reaction (ASR) and carbonation, which will decrease the service life of concrete structures and even cause catastrophic accidents. Repairing and reconstruction of the deteriorated concrete structures will cause damage to the environment, inconveniences in people’s daily lives, and huge economic loss [2].

The durability of concrete refers to the ability to resist deteriorating factors. The harmful consequences would be reduced and even avoided if real-time information on concrete durability could be obtained for guiding daily maintenance and providing timely alarm [3]. Therefore, there is an urgent need for the continuous monitoring of concrete durability throughout the whole lifecycle of concrete structures. Structural health monitoring (SHM) was proposed and widely applied in monitoring concrete durability-related parameters [4].

The sensor is the key unit in the SHM system to provide condition-related data for analysis [5]. Electrical sensors [6] based on the impedance change have the disadvantages of fragility to corrosion, instability, and limited sensing range [7]. Fiber optic sensors (FOS) are made of high-purity silica, which are immune to chemical attacks and electromagnetic interference. These properties make it ideal for long-term durability monitoring of concrete, especially in harsh environments. The characteristics of lightweight, small size, and remote and distributed sensing capability qualify the FOSs for monitoring large-scale concrete structures. However, there is still a gap between the laboratory tests and field applications of concrete durability monitoring. The advantages and disadvantages of FOS need to be comprehensively analyzed, and future research paths also need to be discussed to achieve FOS-based smart concrete.

The purpose of this review is to systematically summarize the recent progress and potential applications of FOS in monitoring durability-related parameters of concrete. The pros and cons of each type of sensor are thoroughly analyzed. Potential research paths in the future are also pointed out. The objectives of this work focus on the durability monitoring for concrete including relative humidity (RH), temperature, chloride ion penetration, corrosion, and pH. High humidity will accelerate concrete carbonation and steel corrosion, which will decrease the service life of the concrete structures. Massive heat is produced in the process of concrete hydration, which leads to thermal cracks, especially for mass concrete. Thus, it is important to monitor the temperature of concrete during and after the pouring process to avoid thermal cracking or other effects on the hydration process. Chloride ions from de-icing salts or marine environments will activate the depassivation of the passive film and accelerate the corrosion process of the steel bar in the reinforced concrete [8,9]. Mass loss of cross-sections is a crucial parameter for evaluating the remaining load capacity of corroded steel rebar and aging concrete structures. A Decrease in pH inside the concrete caused by a chemical attack, acid attack, or carbonation reaction will destroy the passive film on steel and increase the corrosion rate of steel members. pH is thus a significant indicator for evaluating concrete durability. The durability of the concrete constructions could be estimated accurately with continuous monitoring of the above five durability-related parameters.

FOS can be embedded in or attached to the concrete to provide durability sensing capability for smart concrete. However, the sensing principle, packaging methods, sensing characteristics, and deployment of each sensor vary dramatically. Therefore, the novelty of this work is to categorize, compare and discuss the main FOS-based durability monitoring technology including fiber Bragg grating (FBG), long-period fiber grating (LPFG), surface plasmon resonance (SPR), fluorescence-based sensors, and distributed fiber optic sensors (DFOS). This review is supposed to provide recent technical developments and practical applications of durability monitoring for future smart concrete.

## 2. Fiber Bragg Grating (FBG) Sensor

FBG sensors are the earliest developed and most frequently used FOS in civil engineering because of their advantages of low cost, multiplexing capacity, small dimensions, and easy installation [10]. There are several reviews that discussed the applications of FBG sensors in measuring properties including strain, displacement, and micro-vibration induced by ultrasonic waves [11]. This review focuses on the development of FBG-based durability monitoring in recent years.

### 2.1. Sensing Principle

**Figure 1:**
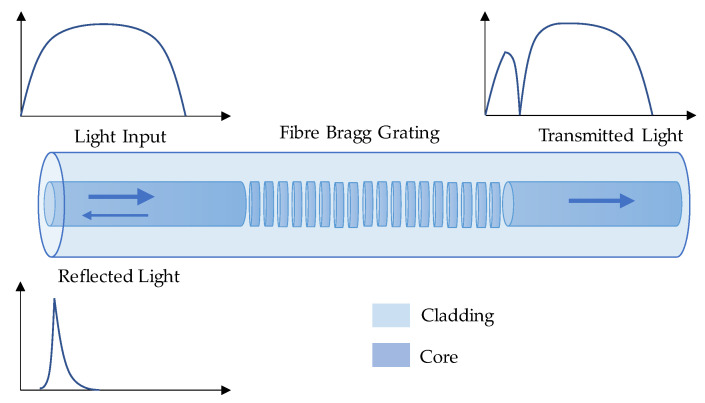
Working principle of an FBG sensor.

As shown in Figure 1, an FBG sensor contains a periodic modulation of the refraction index in the fiber core, which acts as a narrowband filter for the incident light. The grating period is usually in hundreds of nanometers. The reflected central light wavelength shifts with the variations of strain or temperature change and could be utilized for indirect durability monitoring.

In an FBG sensor, the relationship between the reflected central Bragg wavelength λ, strain change Δε, and temperature variation ΔT can be expressed as:

(1) $$\frac{\Delta \lambda }{\lambda }=\left({1\;-\;p}_{eff}\right)\;\Delta \varepsilon +(\alpha +\xi )\;\Delta T$$

where Δλ is the shift of central wavelength, $p_{eff}$ is the photo-elastic parameter related to the fiber property, α is the thermal expansion coefficient, and ξ is the thermal-optic coefficient of the fiber core [12].

### 2.2. Sensor Fabrication

**Figure 2:**
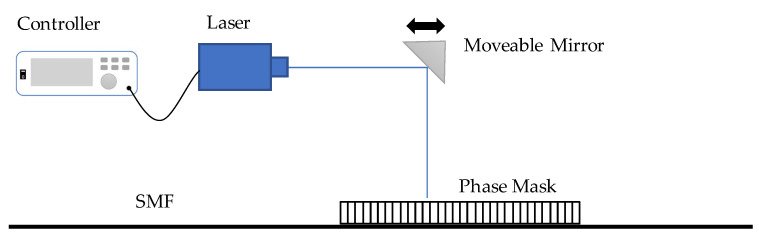
Schematic diagram of the FBG fabrication system [13].

The basic setup for FBG fabrication is shown in Figure 2. The laser beam is reflected by a mirror and irradiates through a phase mask, which is placed parallel to the singlemode fiber (SMF). The mirror can move linearly so that the laser beam can produce nanometer-level grating on the FBG sensor within a designed period [13]. After laser grating, the FBG sensor needs to be packaged. Metal material such as steel is mostly used for sensor fabrication and protection. For durability monitoring, plastic or polymer packaging has also been developed recently to prevent corrosion.

### 2.3. FBG-Based Relative Humidity (RH) Monitoring

*RH* is the ratio of the partial pressure of water vapor in the air–water mixture at a given temperature, which can be defined as:

(2) $$RH=\frac{p_{H_{2}O}}{p_{{sH}_{2}O}}$$

where $p_{H_{2}O}$ is the partial pressure of water vapor and $p_{sH_{2}O}$ is the saturated water vapor pressure at the given temperature.

**Figure 3:**
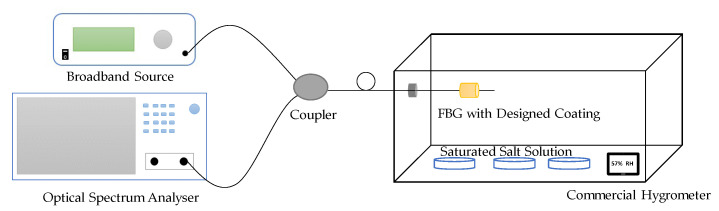
Experimental setup for an FBG humidity sensor [14].

Since all optical phenomena of FBG occur inside the fiber core, FBG itself does not have the capability of sensing *RH*. However, if a humidity-sensitive material can be coated on FBG, the material will swell or shrink with *RH* change, and then FBG could be utilized as an *RH* sensor because it can monitor the swell/shrink strain under different humidity conditions [14]. Figure 3 shows the typical experimental setup in the lab for the FBG humidity sensor. Saturated salt solution is used to produce a stable *RH* environment and a commercial hygrometer is employed to detect the *RH* inside the box.

The developed FBG RH sensors are shown in Table 1. It can be found that agar and polyimide are the most widely used humidity-sensitive coating materials. The FBG-based RH sensor with agar coating developed by Massaroni [15] possessed the highest sensitivity of 0.14 nm/1% RH. The relative humidity obtained from the coated FBG RH sensor could provide RH information and compensate for the FOS-based smart concrete sensing system. For in situ application, some researchers have packaged the sensor and embedded it in concrete [16], and the test results match with the lab experiment. However, the size of the packaged sensor is bulk and needs to be miniaturized in future studies.

### 2.4. Temperature

FBG has been developed as a temperature sensor due to its small size and thermal invasion. Table 2 shows the sensing sensitivity and range of previous research. One limitation of a typical FBG sensor for temperature monitoring is the relatively low sensitivity. To tackle this problem, twin-core fiber [25] and Cu coating [26] were proposed to enhance the sensitivity of FBG temperature sensors. The maximum measuring range of the typical FBG temperature sensor is about 300 °C [27], which also impedes the wide application of FBG temperature sensors. The double-metal layer coating [28], Sn-doped, and $H_{2}$-loaded FBG [29,30] were further investigated to enable FBG sensors for monitoring high temperatures. And the maximum measuring range could reach higher than 500 °C. The high-temperature FBG sensor-based smart concrete is a very promising tool for monitoring the hydration heat of mass concrete (dam and foundations of high buildings, etc.) because it could monitor very large areas using only a single fiber embedded into the concrete.

### 2.5. Corrosion

**Figure 4:**
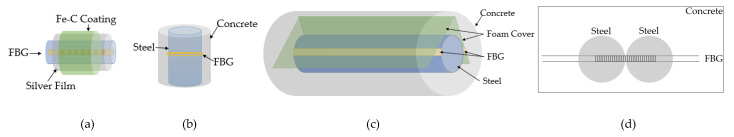
Setup for the FBG corrosion sensor: (**a**) Fe-C-coated [37], (**b**,**c**) binding on rebar [38,39,40], (**d**) notch to deploy FBG [41].

Steel rebar corrosion is one of the most critical issues for the durability of reinforced concrete. Compared to the conventional strain gauge, immunity to chemical attacks and stability make FBG an ideal sensor for corrosion monitoring. The principle of the FBG corrosion sensor is to measure the corrosion-induced strain. There are three main methods to do so: one scheme is to deposit the FBG surface with a Fe-C film, which has similar corrosion properties to steel rebar. As shown in Figure 4a, the longitudinal displacement of the Fe-C coating induced by the corrosion reaction will cause corresponding strain on FBG, which will shift the central wavelength and can be obtained by an optical spectrum analyzer (OSA) [37]. The second method is binding FBG around [38,39] or along [40] the steel rebar, as shown in Figure 4b,c. The third working principle of the FBG corrosion sensor is shown in Figure 4d; a notch was created on the rebar end surface to deploy the FBG sensor, and the relative corrosion-induce strain between two rebars can be measured [41]. FBG corrosion sensor-based smart concrete would have a promising future in field applications if the sensor could be protected well, especially in the pouring process of concrete.

### 2.6. Discussions on FBG-Based Concrete Durability Monitoring

Even though many field applications of FBG-based concrete durability monitoring have been conducted and verified its effectiveness [18,19,20,22,23,25,26,33,34], there are still several limitations to applying FBG in concrete durability monitoring:

(1) The FBG sensor itself can only do strain sensing, so all durability-related parameters need to be converted into strain. However, there are external loads on concrete structures that can cause strain, and the FBG sensor will also measure it, which affects the durability monitoring results. The cross-sensitivity among strain, temperature, and other durability parameters needs to be thoroughly considered in field applications.

(2) Sensor design, installation, and protection of FBG is a critical issue for long-term concrete durability monitoring. Although optic fiber is made of high-purity silica, the packaging and coating material are usually not as durable as the fiber itself. The sensor service life for in situ tests must be considered.

(3) The cost of the FBG sensor is acceptable with the advanced fabrication technology, but the interrogator is relatively expensive compared to other electrical-based data acquisition systems. Moreover, the FBG interrogator is usually fragile and has strict requirements on operation temperatures and RH, and another computer is needed for data storage. All these issues limit the application of FBG and other FOS.

## 3. Long-Period Fiber Grating (LPFG) Sensor

Compared to FBG, the LPFG sensor has a larger grating period ranging from 100 µm to 1 mm [42]. The LPFG sensor promotes coupling between the propagating core mode and the co-propagating cladding modes [43]. This coupling effect is determined by the grating period and refractive index of the surrounding medium, which makes the LPFG sensor sensitive to temperature, strain, and the refractive index (RI) of its external environment. Changes in concrete parameters including RH, pH, and chloride content will cause variations in RI, which could be detected by the shift of LPFG resonance wavelength. Therefore, LPFG has better sensitivity in monitoring the durability-related parameters in concrete compared to FBG.

### 3.1. Sensing Principle

**Figure 5:**
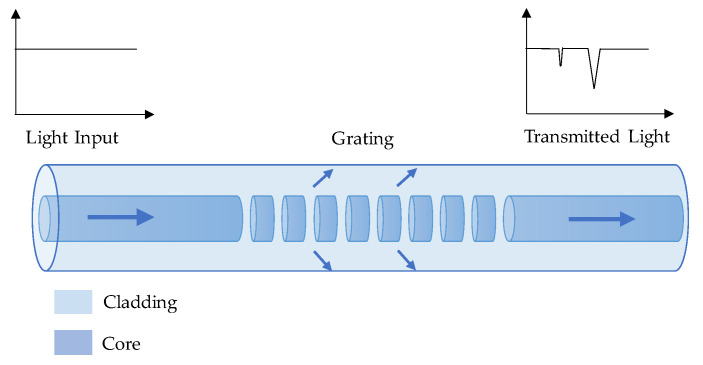
Working principle of an LPFG sensor.

As shown in Figure 5, the working principle of the LPFG sensor relies on coupling the light from the fundamental core mode and the forward propagating cladding modes, which cause several resonant wavelengths to emerge in the output spectrum. The resonant wavelength for i^th^ cladding mode can be obtained by the couple mode theory and phase matching condition, which could be calculated as [44]:

(3) $$\lambda =\left[n_{co}\left(\lambda \right)-n_{cl}^{i}\left(\lambda \right)\right]\Lambda$$

where $n_{co}\left(\lambda \right)$ is the effective RI of the propagating core mode at wavelength λ, $n_{cl}^{i}\left(\lambda \right)$ is the RI of the i^th^ cladding mode, which is partially determined by the RI of the surrounding medium, and Λ is the period of the LPFG sensor.

### 3.2. Fabrication

**Figure 6:**
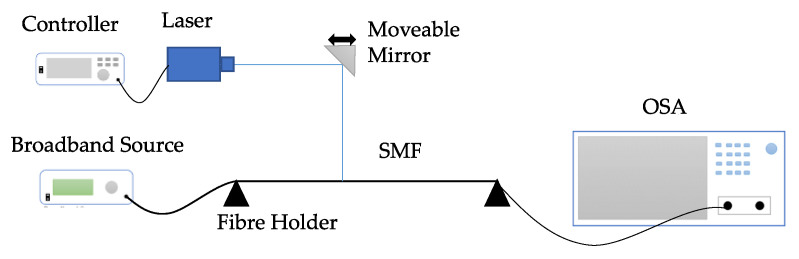
Schematic setup for LPFG fabrication.

As shown in Figure 6, LPFG can be fabricated by the point-by-point grating system. Compared to FBG, the phase mask is not needed for LPFG since its grating period is in hundreds of micrometers. The moveable mirror is applied to change the position of the laser beam; CO_2_ laser is usually the laser source for grating, and OSA is utilized to monitor the LPFG fabrication process.

### 3.3. *RH*

**Figure 7:**
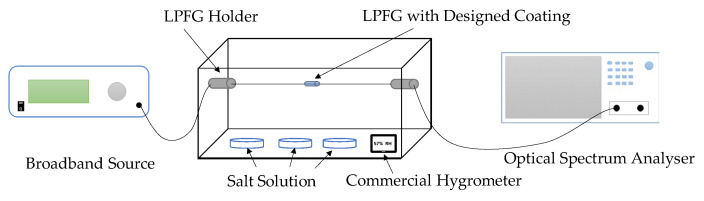
Experimental setup for the LPFG humidity sensor.

The most commonly used experimental setup in the lab to investigate the performance of the LPFG humidity sensor is shown in Figure 7. The salt solution is used to create a certain RH environment. The LPFG sensor is fixed to avoid any bend or strain, which may cause variations in the spectrum. OSA is adopted to obtain the spectrum, and a commercial hygrometer is employed to measure the RH inside. To enhance the sensitivity, an RH-sensitive material is usually coated on the LPFG. However, different from the FBG RH coating, the material used on LPFG does not swell or shrink with RH change. It is the refractive index that changes with different RH levels. So far, the coated LPFG has been extensively explored and shown great potential in RH monitoring.

**Figure 8:**
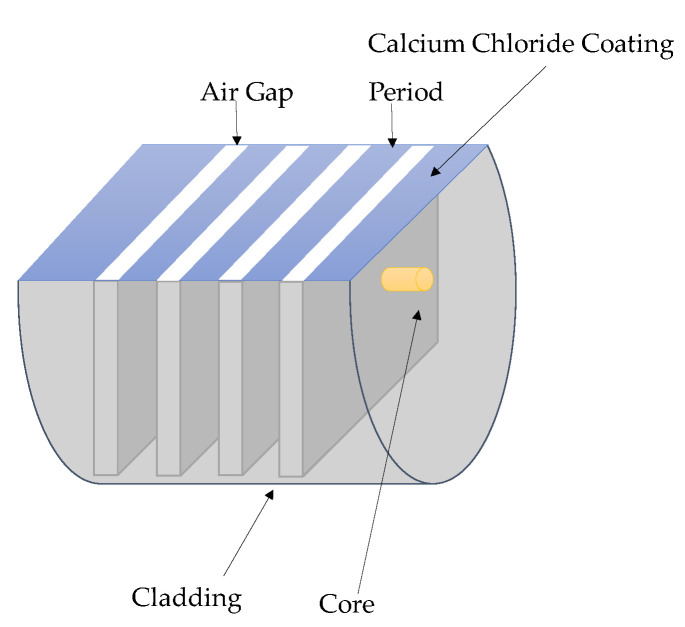
Structure of the LPFG humidity sensor [53].

Polyvinyl alcohol [45,46,47], PAH/PAA [48,49], hydrogel [50], gelatin [51,52], and calcium chloride [53] are the five typical materials used in coating the LPFG for humidity measurement. M. Fu [53] developed an LPFG humidity sensor with a sensitivity of 1.36 nm/1% RH by adopting fiber etching methods to produce an air gap in optical fiber, as shown in Figure 8. A. Theodosiou [54] developed the LPFG sensor in a multimode cyclic transparent optical polymer fiber using the femtosecond laser inscription approach, and the sensitivity could reach 3.3 nm/1%RH. The performance of other developed LPFG humidity sensors is shown in Table 3.

### 3.4. Temperature

Han [60] explored an LPFG sensor to synchronously monitor strain and temperature in 2004, which was the pioneering study of applying an LPFG sensor in temperature monitoring. In this research, LPFG was inscribed on a polarization-maintaining fiber, and the sensitivity of the sensor was 0.0366 nm/°C. Since then, researchers carried out many studies to enhance the temperature sensitivity of LPFG, as shown in Table 4. Four methods including chemical etching, coating, and changing fiber shape and structure have been tried to improve the sensitivity and robustness of LPFG temperature sensors. Among them, the sensitivity of LPFG produced in B–Ge codoped fiber [61] was 2.75 nm/°C, which is the most sensitive among the listed LPFG temperature sensors. In addition, F. Esposito [62] developed an LPFG temperature sensor using panda fiber to simultaneously measure surrounding RI, temperature, and strain by utilizing different sensitivities to the three parameters, which indicates the potential of LPFG in multi-parameter monitoring.

### 3.5. Chloride

Tang [75], Bey [76], and Possetti [77] conducted several pioneering research on LPFG chloride sensors, and the classical experimental setup utilized in their research is shown in Figure 7. The sensitivity of the LPFG chloride sensor in previous research is shown in Table 5. The highest sensing precision of the LPFG chloride sensor could reach approximately 1 ppm, which makes the LPFG chloride sensor a very promising sensing unit in chloride monitoring. But the performance of the LPFG chloride sensor needs to be investigated when the sensor is attached to or embedded in the concrete.

### 3.6. Corrosion

**Figure 9:**
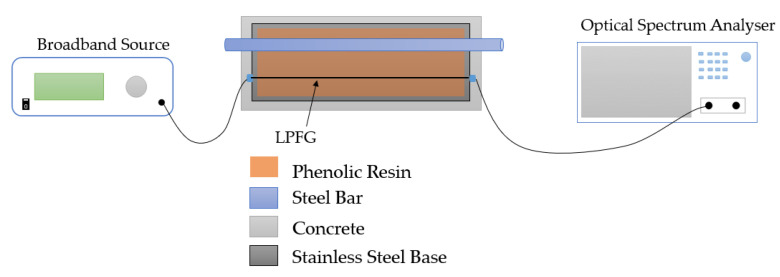
Experimental setup in H. Liu’s research [82].

To monitor the steel bar corrosion inside the concrete in the incipient corrosion stage, an LPFG-based corrosion sensor has been proposed and developed in recent years. H. Liu [82] designed an LPFG corrosion sensor based on phenolic resin, as shown in Figure 9. The dissolution of solid rust-induced RI change in phenolic resin will lead to the shift of LPFG wavelength, which would be obtained by OSA. The corrosion severity could be evaluated by using the corresponding relation between the dust and the shift of resonant peak wavelength.

Fe-C-coated LPFG-based mass-loss measurement is another scheme to monitor the corrosion process since the Fe-C coating has approximately the same chemical component ratio as the steel member or rebar embedded in concrete. Thus, the corrosion process of the Fe-C coating could be utilized to reveal the corrosion process if the SPR fiber sensor was coated with Fe-C and attached to the steel bar.

**Figure 10:**
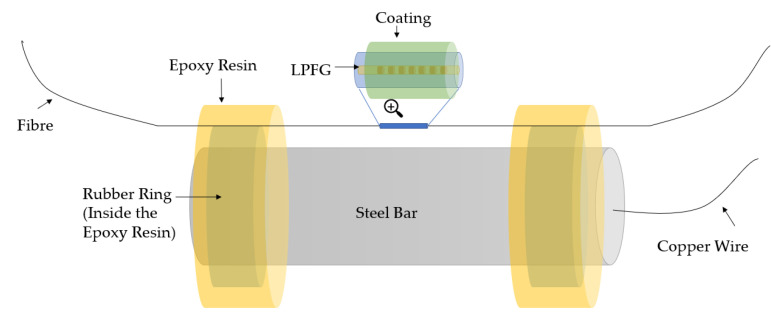
Experimental setup of the Fe-C-based corrosion sensor [83].

Y. Huang [83,84], Y. Chen [85], F. Tang [86,87], and C. Guo [88,89] carried out significant research on Fe-C-coated LPFG-based mass-loss measurement to detect corrosion, and the illustration is shown in Figure 10. Y. Huang [83,84] proposed an LPFG sensor coated with nano iron/silica and polyurethane and established the relation between steel bar corrosion and the nano iron/silica particles by compensating the temperature and pH using a parallel LPFG sensor. In this research, nano iron particles were utilized to promote sensitivity, while the nanosilica particles were designed to facilitate the robustness of the sensor. Y. Chen [85] deposited the LPFG sensor with a 0.8 µm thick Ag and then electroplated the LPFG sensor with a 20 µm thick Fe-C. The calibrating experiments showed that the resonant wavelength of the sensor decreases in the process of steel bar corrosion and the sensitivity of the LPFG corrosion sensor was considerably improved. However, there is a trade-off between the service life and corrosion sensitivity in designing an Fe-C-coated LPFG corrosion sensor. To improve the sensitivity and service life of the Fe-C-coated LPFG sensor, C. Guo [88,89] coated the LPFG sensor with a Gr/AgNW composite, and the results demonstrated that both sensitivity and service life of the Gr/AgNW-coated LPFG corrosion sensor were significantly improved compared with Ag inner coating.

### 3.7. pH

The high alkalinity of concrete prevents the embedded steel rebar from corrosion by forming a passive film on the rebar surface. The decrease in pH caused by chloride ion ingression, acid attack, and carbonation reaction may lead to steel corrosion and concrete spalling, which makes pH a significant parameter for concrete durability. The traditional pH measurements, including chemical indicators and in situ leaching methods [3], are destructive to concrete. The LPFG sensor coated with pH-sensitive hydrogel provides an in situ and non-destructive manner for pH monitoring. The performance of the developed LPFG pH sensor in recent years is listed in Table 6.

### 3.8. Discussions on LPFG-Based Durability Monitoring

Compared to FBG, LPFG has the unique capability of sensing the RI of its surrounding medium, which is superior for durability monitoring. However, similar to FBG, LPFG also has limitations in cross-sensitivity, packaging, and interrogator cost. Furthermore, LPFG has its own issues that need to be addressed for smart concrete.

(1) Compared to the phase mask fabrication method of FBG, LPFG fabrication is relatively complicated since it needs CO2 laser point-by-point inscription or acid etching. These fabrication processes usually are expensive in terms of equipment, and the grating accuracy and robustness are difficult to control, which limits LPFG production at the commercial level. A more accurate and efficient fabrication method for LPFG is in urgent need.

(2) Different from FBG, LPFG sensing depends on the transmission spectrum, which means that two ports are needed for LPFG interrogation. This will increase the workload and installation difficulty for LPFG sensors, especially for deployment in concrete. This is also the reason that the above research has not applied the LPFG sensor in real concrete structures. Further study on LPFG packaging and in situ concrete durability monitoring needs to be conducted.

## 4. Surface Plasma Resonance (SPR) Sensor

SPR refers to the resonant oscillation of conduction electrons at the interface between negative and positive permittivity material stimulated by incident light. Light in optical fiber transmits with total internal reflection, which makes the SPR principle of prism coupling suitable for optical fiber [94]. This coupling causes a decrease in the reflected intensity and a resonate trough which is sensitive to RI [95]. R. C. Jorgenson [96] innovatively proposed a fiber optic chemical sensor based on SPR in 1992 and built a theoretical model of the sensor. This theory is the fundament of both singlemode–multimode–singlemode (SMS) sensors and multimode–singlemode–multimode (MSM) sensors.

### 4.1. Sensing Principle

#### 4.1.1. SMS Structure

**Figure 11:**
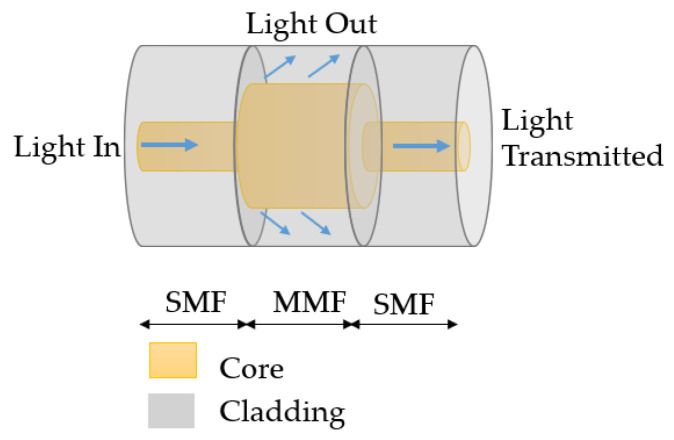
Schematic of an SMS structure.

The SMS structure is a widely used SPR sensor, which is produced by splicing a multimode fiber (MMF) between two singlemode fibers, as shown in Figure 11. The light leading to SMF enters the MMF section and excites guided modes. The guided modes continue to propagate and couple to the core mode of the singlemode fiber (SMF) leading out. The longitudinal propagation constants for the excited modes are related to the cladding RI of the MMF; thus, the spectrum will change with the RI [97].

#### 4.1.2. MSM Structure

**Figure 12:**
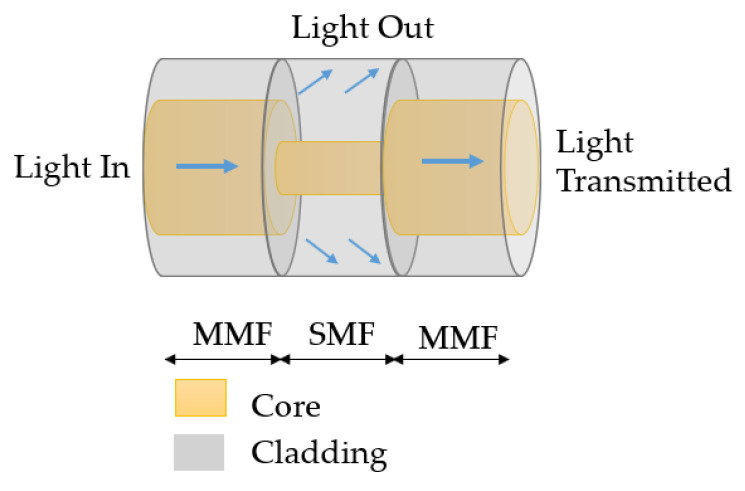
Schematic of an MSM structure.

The MSM structure is produced by splicing a singlemode fiber between two multimode fibers, as shown in Figure 12. When light transfers from multitude mode fiber to singlemode fiber, a part of the light is coupled from multitude mode fiber core to singlemode fiber cladding. Similar to the SMS structure, the coupling will cause a trough in the spectrum, which will change with the variations of the RI.

### 4.2. Fabrication

The fabrication procedures of the SMS structure are as follows: the cladding of SMF is first stripped, the MMF is intercepted to the specified length, and the fibers are spliced in sequence with splicer [98]. The fabrication procedures of MSM are similar to SMS.

### 4.3. RH

**Figure 13:**
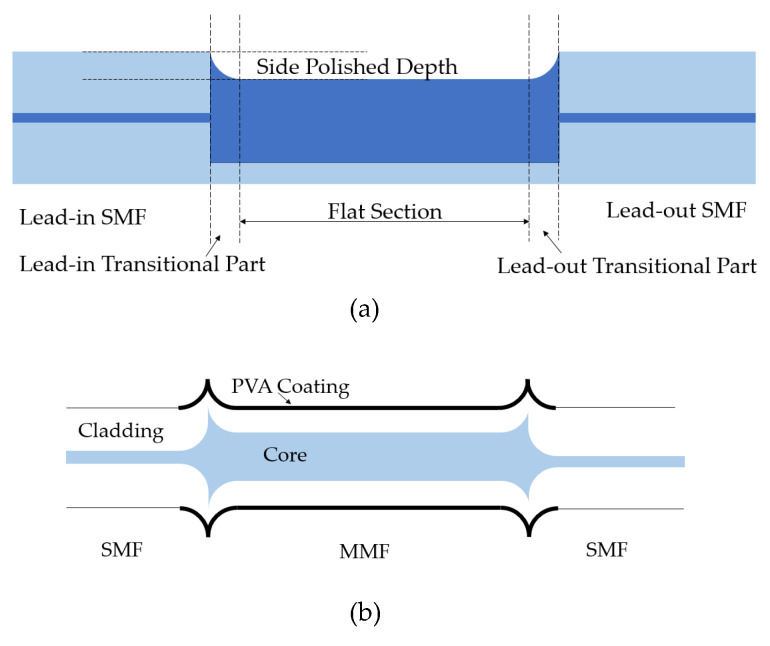
(**a**) Polishing [100] and (**b**) coating [102] applied in an SMS humidity sensor.

Various setups can be applied in the SMS humidity sensor to improve the sensitivity. J. An [99], X. Wang [100], W. Xu [101], and H. Lin [102] conducted several interesting studies on designing various structures to improve the sensitivity and robustness of SMS humidity sensors. J. An [99] developed an SMS fiber structure with two waist-enlarged tapers, which makes this structure possess higher robustness compared with a typical SMS fiber structure, as shown in Figure 13a. The developed sensor has a sensitivity of 0.223 nm/1% RH. X. Wang [100] designed a side-polished SMS fiber humidity sensor, which was polished by a rolling wheel overlaid with abrasive paper, as shown in Figure 13b. The sensitivity of this sensor is 0.14 dB/1% RH. H. Lin [102] designed a spiral micro-structured SMS fiber structure coated with PVA, and the RH sensitivity of the sensor is 0.650 nm/1% RH.

### 4.4. Temperature

The SPR temperature sensor has attracted much attention and has been widely developed. Researchers adopted four methods including chemical etching, coating, changing shapes of fiber, and altering structures to enhance the sensitivity of SPR fiber sensors to temperature.

**Figure 14:**
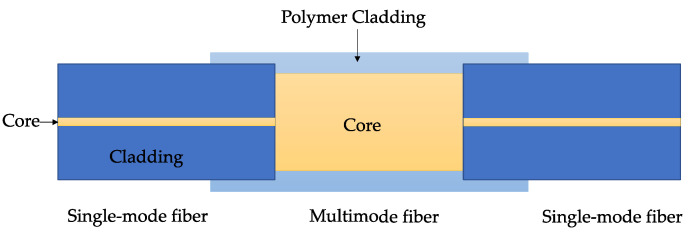
Structure of a temperature sensor; Y. Zhang [103].

**Figure 15:**
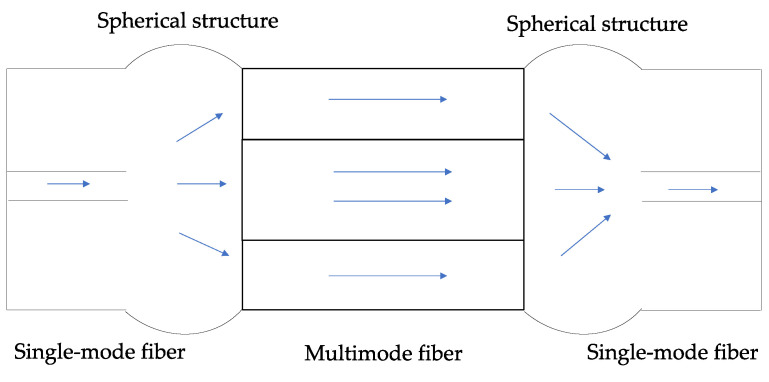
A schematic diagram of a spherical structure; J. Su [104].

It can be seen in Table 7 that the temperature sensitivity of the SPR sensor is around 0.1 nm/°C. Cladding has shown to be effective in enhancing the sensitivity of the SPR sensor to temperature, and a typical example is shown in Figure 14 [103]. In this research, Y. Zhang adopted thermo-optic polymer cladding to enhance the temperature sensitivity of SMS fiber sensors, and the sensitivity reached 15 nm/°C. Adopting different shapes and structures is also effective in improving temperature sensitivity. J. Su [104] developed a temperature sensor with a spherical structure, and the sensitivity was 0.131 nm/°C, as shown in Figure 15.

### 4.5. pH

pH-sensitive hydrogel coating was widely used in SPR pH sensors for providing an in situ and non-destructive manner to monitor pH. Table 8 shows the performance of the recently developed SPR pH sensor.

### 4.6. Discussions of the SPR Sensor for Durability Monitoring

Even though SPR sensors have been used in biological monitoring, there are few in situ applications in civil engineering, such as embedding in concrete for durability monitoring. Similar to LPFG, one of the main obstacles impeding the SPR sensor to wider application is the complex fabrication procedure. The SMS or MSM structures make it hard to achieve standard and efficient production. That is why most research is still in the lab stage.

## 5. Fluorescence-Based FOS

### 5.1. Sensing Principle

The fluorescence-based fiber optic sensor is a simple but effective inspector in concrete durability monitoring because of the unique working principle and capacity of on-field and real-time monitoring [120]. The widely employed fluorescent materials include high molecular organic fluorescent groups, fluorescent dyes, and semiconductor quantum dots, of which color varies reversibly upon a change in ion concentration [121].

**Figure 16:**
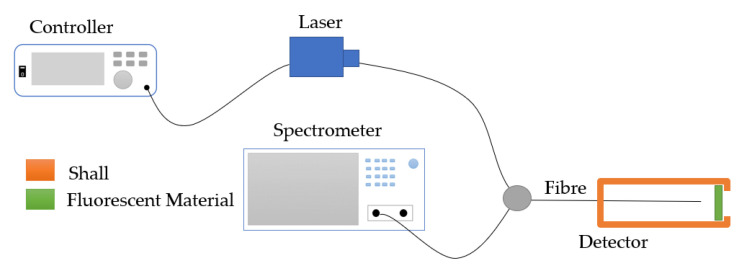
Setup of a fluorescence-based optical sensor [122].

The basic setup in the lab for the fluorescence-based sensor consists of a laser source, a Y-type optical fiber, an optical fiber spectrometer, and a detector containing fluorescent material, as shown in Figure 16. The laser controller adjusts the wavelength of the laser source to excite the fluorescent material in the designated detector [122].

### 5.2. Temperature

**Figure 17:**
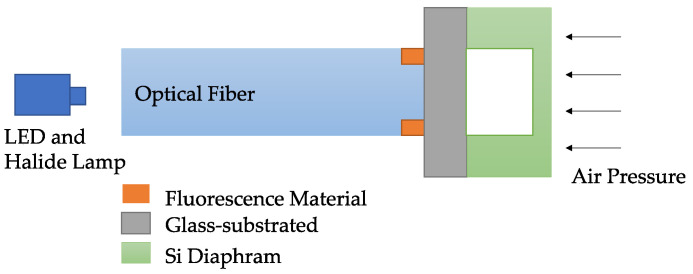
Setup of a fluorescence-based hybrid fiber-optic sensor [126].

Fluorescence-based temperature sensors utilize a single fiber to transmit both excitation and emission signals. The advantage of self-calibrating [123] attracts the interest of many researchers. The characteristic of tunable fluorescence emission under temperature variations can be employed in fluorescence-based temperature sensors. For example, the emission intensity of ${\left\{\left[\mathrm{Cd}\left(\mathrm{TBAPy}\right){\left(H_{2}O\right)}_{2}\cdot {4(H}_{2}O)\right]\right\}}_{n}$ decreases linearly, with the temperature increasing from 33.8 °C to 148.8 °C [124], and the emission intensity of ${\mathrm{Cu}}_{5}\;\mathrm{metal}\;\mathrm{clusters}$ drop linearly with a temperature increase from −45 °C to 80 °C [125]. As shown in Table 9, Lophine, phosphor, and fluorescent dye 10(3-sulfopropyl) acridinium betaine were applied in a fluorescence-based temperature sensor with the setup shown in Figure 16. Jiang [126] developed a hybrid fiber optic sensor that can simultaneously measure the temperature and pressure, and the setup is shown in Figure 17. It can be seen in Figure 17 that the F-P cavity and fluorescent material can provide two independently reflected lights, which can be transmitted to the pressure and temperature separately.

### 5.3. pH

Many fluorescence-based pH sensors have been explored and developed in recent years. As shown in Table 10, the large number of pH-sensitive fluorescent materials utilized in optical sensors are limited in sensing pH range. The sensing range of fluorescent materials in previous research was near the neutral pH region [128,129,130,131,132,133,134,135], which might be not suitable for concrete durability monitoring with high alkalinity. In recent research, researchers tried to develop fluorescence-based pH sensors to measure pH values in the range of 10–13. It can be seen in Table 11 that azo dye [136], thymol blue [137], coumarin imidazole dye [138], and Naphth-AlkyneOMe entrapped in a cross-linked polyvinyl alcohol–glutaraldehyde matrix [139] could be applied to measure pH values in the range of 10–13. Among these materials, coumarin imidazole dye had the most suitable measuring range to build smart concrete for pH monitoring in civil structures.

### 5.4. Discussions on the Fluorescence-Based Optical Sensor for Durability Monitoring

Compared to the previous FOS, the advantage of a fluorescent sensor is that it has ion selectivity, which means the sensor can differentiate chloride, sulfate, OH, and other ions. This is crucial for durability monitoring since different ions have different effects on concrete. However, the service life of fluorescence-based optical sensors is limited by the short life span of the fluorescence material, which might make it fail to satisfy the requirement of long-term concrete durability monitoring. The main reason is that the fluorescent material will deteriorate; thus, the fluorescent intensity will decrease with time.

## 6. Distributed Fiber Optics Sensors (DFOS)

### 6.1. Sensing Principle

The sensing principle of distributed fiber optic sensing is sending a laser pulse along a fiber optic cable and then measuring the laser that is reflected or backscattered. There are three types of backscattering used for measurement: Raman, Brillouin, and Rayleigh [140].

#### 6.1.1. Principle of Raman Backscattering in Distributed Fiber Optic Sensing

Raman backscattering is mainly used in DTS (distributed temperature sensing). A DTS instrument sends a short laser pulse along a fiber optic cable and measures the laser scattered back in the fiber cable. Most backscatter has the same wavelength as the original laser pulse. The other backscatter shows the effect of Raman backscattering. The DTS instrument measures the intensity of the two backscatters, Stokes scatter and anti-Stokes scatter, of which Stokes scatter has a longer wavelength and anti-Stokes scatter has a shorter wavelength. The intensity of the anti-Stokes scatter is sensitive to the temperature [141].

#### 6.1.2. Principle of Brillouin Backscattering in Distributed Fiber Optic Sensing

Brillouin backscattering is a basic physical process representing the interaction effect between light and optical medium in a propagation medium. Brillouin optical time domain reflectometry (BOTDR) and Brillouin optical time domain analysis (BOTDA) are two commonly used distributed fiber optic sensing based on Brillouin backscattering [142].

**Figure 18:**
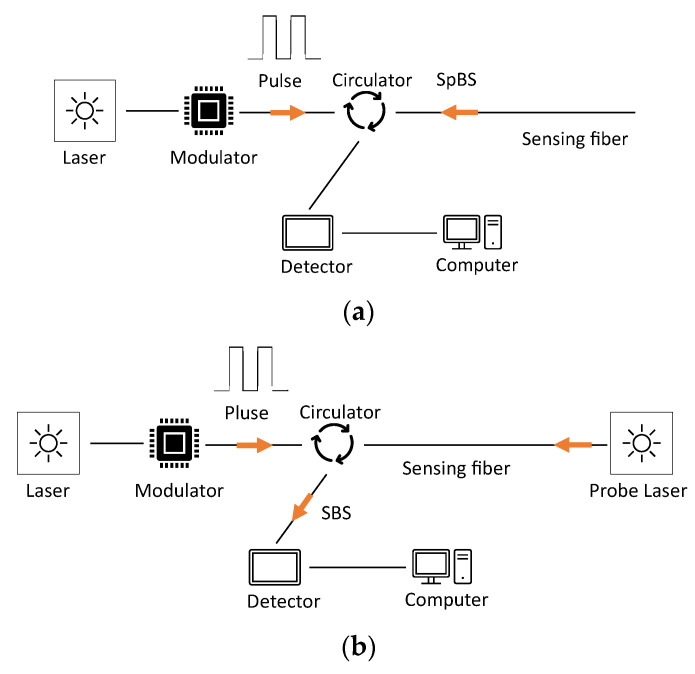
Sensing principle of (**a**) BOTDR and (**b**) BOTDA [142].

BOTDR is a single-ended access technology, and it is based on spontaneous Brillouin scattering (SpBS). When an incident light propagates forward, it will produce spontaneous Brillouin backscattered light along the fiber, as shown in Figure 18a. The frequency of the backscattered light changes linearly to the change in pressure, temperature, and strain along the optical fiber cable [142,143].

BOTDA is a more complicated form of BOTDR, and it is based on the Stimulated Brillouin Scattering (SBS) effect. Figure 18b shows the setup of the BOTDA-based sensing system. Light signals, including a short pump pulse light and a probe light, are injected into two ends of an optical fiber cable. Then, Brillouin scattering is stimulated when the frequency difference meets the local Brillouin frequency of the optical fiber cable. The use of both pump pulse light and probe light at two ends of the optical fiber cable aims to enhance the Brillouin scattering process. The obtained frequency shift of the Brillouin gain spectrum is sensitive to the change in temperature and strain along an optical fiber cable [142].

#### 6.1.3. Principle of Rayleigh Backscatter in Distributed Fiber Optic Sensing

Optical frequency domain reflectometry (OFDR) operates based on Rayleigh backscattering, which is caused by the interactions between the fluctuations in the density of the fiber optic core and the light. The fiber cable extends or contracts with the change in temperature or strain. Then, the distance between the imperfections in the fiber cable changes and will cause a change in the frequency of the backscattered light [144].

### 6.2. Corrosion

Distributed fiber optic sensors can provide information along the entire fiber length, which makes this technology suitable for monitoring corrosion in long lengths [145]. In many studies, distributed sensors have been applied to monitor corrosion along steel bars and in most cases, these corrosion sensors are based on the measurement of strain changes conducted by the expansion of corrosion products.

**Figure 19:**
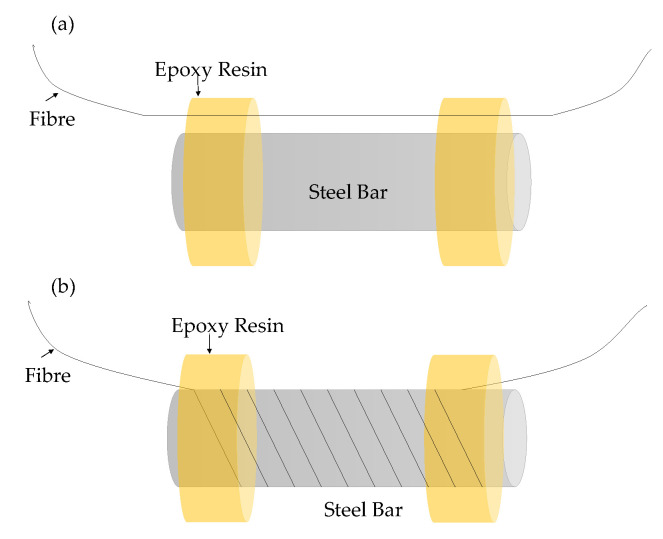
DFOS deployment for corrosion monitoring: fiber (**a**) along and (**b**) around steel rebar [146,147,148].

As corrosion sensors, the Brillouin backscattering distributed fiber optic sensors are installed mainly in two methods. The first method is to tightly install a singlemode optical fiber around a steel bar [146,147], as shown in Figure 19a. The second method is to install the optical fiber along the steel bar [148], as shown in Figure 19b. Both methods aim to monitor the strain changes caused by the volume expansion of the corrosion product. In recent research, pulse-pre-pump BOTDA (PPP-BOTDA) technology was applied, which achieved a higher spatial resolution [145,148]. The special resolution increased from 1 m [146] to 2 cm [148]. Even though DFOS is a promising method for corrosion monitoring since it can provide corrosion-induced strain over long distances, the monitoring results may be coupled with the mechanical strain caused by external loads. On the other hand, the installation of DFOS on steel rebar is more complex than point FOSs.

Apart from Brillouin backscattering, Optical frequency domain reflectometry (OFDR) is another widely used distributed fiber optic sensing technology used in corrosion monitoring. The OFDR technology is applied mainly in three methods. The first method is close to the application of Brillouin backscattering distributed fiber optic sensor in corrosion monitoring. In this method, a singlemode optical fiber cable is tightly attached to the surface of the steel bar to monitor the strain changes caused by the volume expansion of the corrosion product [149,150].

### 6.3. Shrinkage and Crack

**Figure 20:**
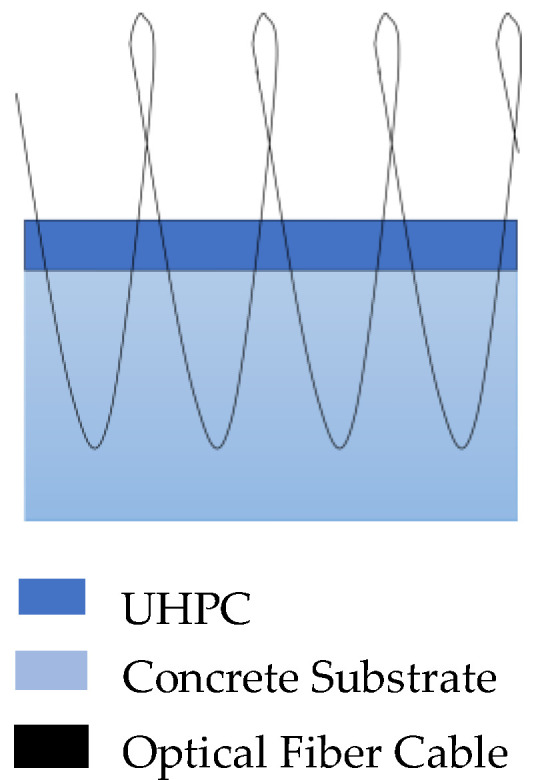
DFOS setup for monitoring delamination between UHPC and concrete [153].

Cracks before the concrete gets loaded are mainly induced by the differential and excessive shrinkage [151]. To gain a distributed measurement of shrinkage, distributed optical fiber sensing has been used in some studies. Bao et al. [152] applied pulse-pre-pump BOTDA technology to measure strain induced by early age shrinkage of mortar. The average shrinkage measured by PPP BOTDA agrees with the average measured in the standard test. Additionally, Bao [153] cast 450 mm (length) × 200 mm (width) × 25 mm (thickness) UHPC overlays over an existing 200 mm thick concrete substrate and installed PPP BOTDA sensors inside the samples. As shown in Figure 20, the optic fiber was installed in a serpentine manner between the UHPC and concrete to monitor the delamination behavior. The results show that the PPP Brillouin distributed sensor can detect initiation and propagation of delamination due to the early age shrinkage of the UHPC overlay. Rayleigh backscatter-based distributed fiber optic sensors have been applied in shrinkage monitoring as well. For example, Yager [154] developed a DFOS-based shrinkage monitoring system in large-scale deep beams and one-way slab strips. The results show that for ordinary Portland cement concrete, restrained shrinkage strains were uniformly distributed. Other research has also been conducted using DFOS to monitor the cracks [155,156] and bond performance [157] of the concrete.

### 6.4. Discussions on Distributed Fiber Optic Sensing-Based Durability Monitoring

Distributed fiber optic sensors have the unique capability of providing distributed strain measurements along the whole length of the fiber optic cable, which makes them ideal for monitoring the durability of large structures. Additionally, the distributed sensors are cheaper compared with FBG and LPFG since grating fabrication is not needed. Brillouin and Rayleigh are the main two backscatterings used for measurement in durability monitoring. The sensitivity and monitoring range can be increased with the optimization of the transfer coefficient between the volume expansion of corrosion products, shrinkage, or crack-induced strain and the fiber cable.

## 7. Conclusions

In this review, the sensing principle, fabrication methods, durability monitoring capability, advantages, and disadvantages of five main types of FOS are comprehensively summarized. The limitations of each sensor are discussed. In general, different types of FOS have their own advantages in specific parameter monitoring. LPFG and SPR sensors showed superiorities in RH, chloride, and corrosion monitoring since they possess high sensitivity and a larger sensing range for RI. FBG sensors have been widely used in strain and temperature monitoring. In terms of ion and pH monitoring, the fluorescence-based sensor is a promising technology due to its unique property of ion selectivity. The DFOS sensor has a unique advantage in long-distance monitoring for large-scale concrete structures compared to other FOS, especially for corrosion, shrinkage, and crack monitoring.

In recent years, lots of research has been carried out to develop a durability monitoring sensor using optic fiber. The concept of smart concrete has thus been proposed since the FOS can provide critical information for daily maintenance, increasing the service life and avoiding possible catastrophic accidents. To further achieve smart concrete, the FOS still has several issues that need to be addressed, and these might provide research insights for future study.

(1) Fabrication: the FBG and LPFG sensors are fabricated through laser inscription. SPR sensors are fabricated through the splicing of single- or multimode fiber or side-polishing. A fluorescent sensor needs to synthesize the chemical material. So far, only the FBG sensor fabrication has achieved the commercial level for high-efficiency production. The other types of sensors are still fabricated in the lab in limited quantities. This is the reason that we only see FBG sensors embedded in concrete for in situ monitoring. The next step of FOS development needs to improve the fabrication efficiency to meet a large amount of sensor demand for smart concrete.

(2) Packaging: even though FOS has advantages in size and sensitivity and is immune to electromagnetic interference, it is fragile under tension, bending, or impact. To make the sensor stable and durable, packaging is the most essential procedure before application. However, the packaging material itself also needs to be durable. Steel or plastic might not be suitable for harsh environments such as corrosion, UV light, and high temperatures. Ceramic has also been used for sensor packaging in recent years, but its ductility is relatively low. Therefore, an ideal packaging material is urgent for FOS. On the other hand, the packaging size also matters. The mechanical manufacturing of steel or plastic has difficulty when the required size is small or irregular. Three-dimensional printing is a very promising path in this field.

(3) Multi-parameter monitoring: the durability of concrete involves many parameters such as crack width, moisture, pH, corrosion, and temperature. If the FOS can only monitor one parameter, the sensing system, connection wires, and data acquisition module will be so bulky that it is difficult for engineers to manipulate. By utilizing the difference in sensitivity, wavelength multiplexing, and advantage in small size, FOS has the potential to achieve multi-parameter monitoring, which can not only simplify the sensor installation and administration but also reduce the average cost of the entire system since one interrogator can provide more data.

(4) Distributed sensing: the point FOS is so small compared to the concrete structures, especially long-span bridges, long-distance tunnels, and tall buildings. One way to address this issue is using a sensor array. By connecting lots of FOS in one loop, or fabricating hundreds, even thousands of sensors (grating or SPR) on one optic fiber, distributed sensing of large-scale concrete can be achieved. However, the sensor array path requires a high-performance interrogator for data acquisition. Another strategy is utilizing distributed fiber optic sensors (DFOS). As discussed in Section 6, DFOS is based on the Rayleigh, Brillouin, or Raman scattering effect, which can monitor strain and temperature in millimeter-level spatial resolution. Due to its unique capability in long-distance (maximum 20 km so far) monitoring, DFOS has attracted more attention in the past decade for concrete shrinkage, crack, and corrosion monitoring. Nevertheless, DFOS right now is still in the temperature and strain sensing stage. Parameters such as pH, ion concentration, and moisture cannot be monitored by DFOS so far. This should be part of the DFOS development in the future for smart concrete.

(5) Concrete embedment: to the best of our knowledge, not all types of FOS discussed in this work have been embedded in concrete for in situ monitoring. The main problem in real applications is that some sensors or optic fibers cannot survive during the concrete vibration and consolidation process. As discussed in the packaging section, part of the reason is that the packaging material cannot provide enough protection to the fragile FOS. Moreover, the normal communication optic fiber is commonly used for signal propagation due to its thin coating and small diameter. However, this kind of fiber is fragile to impact, squeeze, and shear force. Once the fiber is broken in one spot, the entire sensing loop is destroyed, even though the sensor itself might be functional. Some may argue that more rugged fiber cables can be used, such as an armored cable with steel or aluminum protection. However, the larger diameter of an armored cable may affect the performance of the concrete. When the fiber cable is attached to the steel rebar, the fiber cable may reduce the adhesion between the concrete and rebar. Therefore, even though FOS shows promising potential in concrete durability monitoring, the deployment of the sensor inside the concrete still needs more effort in both scientific and engineering considerations.

## Figures and Tables

**Table 1:** Performance of the FBG humidity sensor in previous research.

Coating	Sensitivity	Range/1 % RH	Applied in Concrete	Reference
Agar	0.14 nm/1% RH	10–95	No	[15]
Polyimide	0.00297 nm/1% RH	30–90	No	[16]
Polyimide	0.002 nm/1% RH	30–70	No	[17]
Polyimide	0.00338 nm/1% RH	25–95	Yes	[18]
Agar	0.051–0.036 nm/1% RH	40–90	Yes	[19]
Polyimide	0.0056 nm/1% RH	23–97	Yes	[20]
ORMOCER	0.0024 nm/1% RH	11.3–97.3	No	[21]
Di-Ureasil	0.0222 nm/1% RH	15–95	Yes	[22]
Polyimide	0.005 nm/1% RH	20–90	Yes	[23]
Polyimide	0.0045 nm/1% RH	22–97	No	[24]

**Table 2:** Performance of the FBG temperature sensor in previous research.

Coating/Method/Fiber	Sensitivity	Range/°C	Applied in Concrete	Reference
Twin-core fiber	0.0221 nm/°C	27–46	Yes	[25]
Copper coating	0.0148 nm/°C	30–80	Yes	[26]
Hydrogen-loaded FBG	0.0175 nm/°C	around 1000	No	[27,28,29,30]
-	0.0343 nm/°C	30–70	No	[31]
-	0.014 nm/°C	41–90	No	[32]
Tilted Bragg reflector fiber laser	0.01075 nm/°C	31–71	Yes	[33]
Polyurethane–graphene (PU-graphene) nanocomposite	0.006 nm/°C	25–60	Yes	[34]
Superstructure FBG	0.0113 nm/°C	20–110	No	[35]
Moveable flanges	8.45 ± 2.05 pm/°C	−20–80	No	[36]

**Table 3:** Performance of the LPFG humidity sensor in previous research.

Coating	Sensitivity	RH Range (%)	Reference
Polyviny alcohol	0.81 nm/1% RH	53–75	[45]
Polyviny alcohol	0.1723 nm/1% RH	20–80	[46]
Polyviny alcohol	0.07018 nm/1% RH	32–99	[47]
PAH/PAA	0.06323 nm/1% RH	20–80	[48]
PAH/PAA and Al_2_O_3_/PSS	0.04 nm/1% RH	20–90	[49]
Hydrogel coating	0.0099 nm/1% RH	60–100	[50]
Gelatin coating	0.833% RH/dBm	90–99	[51]
Gelatin/cobalt chloride coating	0.18 nm/1% RH	35–90	[52]
Calcium chloride coating and air gap produced by fiber etching methods	1.36 nm/1% RH	55–95	[53]
Multimode cyclic transparent optical polymer	3.3 nm/1% RH	20–40	[54]
Poly(ethylene oxide)/cobalt chloride (PEO/CoCl_2_)	0.185 nm/1% RH	50–77	[55]
Polymide coating and a silver mirror at the end	0.10 nm/1% RH	20–80	[56]
Hydrogel coating (acrylic acid and vinyl pyridine)	0.2 nm/1% RH	38.9–100	[57]
Graphene oxide	0.15 dB/1% RH	60–95	[58]
Silica nanoparticles	0.53 nm/1% RH	35–98	[59]

**Table 4:** Performance of the LPFG temperature sensor in previous research.

Coating/Method	Sensitivity	Range/°C	Reference
-	0.0366 nm/°C	35–90	[60]
B–Ge codoped fiber	2.75 nm/°C	0–60	[61]
Panda fiber	0.0704 nm/°C	20–60	[62]
$Al_{2}O_{3}$ nanofilm	0.77 nm/°C	20–120	[63]
Etched by hydrofluoric acid (HF)	0.4028 nm/°C	20–50	[64]
-	0.31 nm/°C	25–125	[65]
Secondary modulated tapered fiber	0.065 nm/°C	30–90	[66]
Poly-dimethylsiloxane (PDMS)	0.255.4 nm/°C	20–80	[67]
-	1.63 nm/°C	22–52	[68]
-	0.085 nm/°C	Δ*T* = 60	[69]
Two LPFG hybrid systems	0.04 nm/°C	20–120	[70]
LPFG in a heat-shrinkable tube	0.15 nm/°C	−20–50	[71]
-	0.07 nm/°C	20–100	[72]
Cascaded optical fiber	0.04587 nm/°C	20–80	[73]
LPFG and liquid-filled PCF	0.0642 nm/°C	23–68.9	[74]

**Table 5:** Performance of the LPFG chloride sensor in previous research.

Coating	Sensitivity	Reference
Gold colloids	0.071 nm/1% NaCl	[75]
-	0.02848 nm/ppm	[78]
-	1.26 nm/1% NaCl	[79]
-	0.00661 nm/g $\cdot L^{-1}$(NaCl)	[77]
Hydrogel	7 nm/M (125.5 pm/‰)	[80]
Polyelectrolyte	36 nm/M	[81]

**Table 6:** Performance of the LPFG pH sensor in previous research.

Coating	Range	Response Time	Sensitivity	Reference
PB in the PAH/PAA	4–7	60s	28.3 nm/pH	[90]
Hydrogel	2–12	2s	0.66 nm/pH	[91]
Hydrochloride and polyacrylic acid	4–7	120s	28.3 nm/pH	[92]
Polyelectrolyte-wrapped multi-walled carbon nanotubes	2–13	-	0.83 dB/pH	[93]

**Table 7:** Performance of the SPR temperature sensor in previous research.

Coating/Method	Sensitivity	Range/°C	Reference
-	0.172 nm/°C	65	[95]
Liquid-sealed coreless multimode fiber	5.15 nm/°C	10–30	[97]
SMS-ULPFG structure	0.04485 nm/°C	26–118	[105]
Spherical structure	0.131 nm/°C	20–70	[104]
Spherical-shaped structures	0.1193 nm/°C	25–735	[106]
Polymer cladding with a large negative thermo-optic coefficient	3.195 nm/°C	28–39	[107]
-	0.016 nm/°C	20–50	[108]
No-core fiber	0.092 nm/°C	30–95	[85]
-	0.01733 nm/°C	15–75	[109]
Polymer (PMMA) plate frame	6.5 nm/°C	51–65	[110]
Polyurethane–acrylate	15 nm/°C	72–78	[103]
Half-tapered fiber	0.08958 nm/°C	25–50	[111]
Pure silica multimode fiber	1.88 nm/°C	0–25	[112]
-	0.060 nm/°C	30–150	[113]
Tapered structure	0.0878 nm/◦C	30–55	[114]
Compact strain-insensitive fiber	0.05279 nm/°C	30–90	[115]
-	0.0582 nm/°C	20–70	[116]
-	0.04412 nm/°C	10–70	[117]

**Table 8:** Performance of an SPR pH sensor in previous research.

Coating	Range	Response Time	Sensitivity	Reference
Hydrogel	1–12	24s	13 nm/pH	[94]
pH-responsive poly hydrogel	6.75–8.25	55s	1.71 nm/pH	[118]
Precursor TEOS and pH-sensitive indicators	1–13	40s	0.6 dBm/pH	[119]

**Table 9:** Performance of the fluorescence-based temperature sensor in previous research.

Coating	Range/°C	Reference
Fluorescent dye 10(3-sulfopropyl) acridinium betaine	30–250	[123]
Phosphor	25–80	[126]
Lophine	5–30	[127]

**Table 10:** Performance of fluorescent material in previous research.

Material	Range	Reference
Pyrene structure attached to methoxypyridine	1.26–3.97 and 8.54–10.36	[128]
Two water-sTable 2-D zinc(II) compounds	7–11	[129]
Hydroxypyrene-1,3,6-trisulfonic acid trisodium salt (HPTS)	5.7–9.0 and 4.2–5.7 and 3.4–4.2	[130]
5(6)-FAM and Porphyrin	5.5–8.0	[131]
Porphyrinic Zr metal—Organic framework	1–11	[132]
Conjugated polymers containing pyridine rings	1–7	[133]
Fluoresceinamine isomer II (FA)	4–10	[134]
Chloro phenyl imino propenyl aniline (CPIPA) and nitro phenyl imino propenyl aniline (NPIPA)	8–12 and 7–12	[135]

**Table 11:** Performance of the fluorescence-based pH sensor in recent research.

Coating	Range	Reference
Azo dye	10.4–12.24	[136]
Thymol blue	11–13	[137]
Coumarin imidazole dye	10–13.2	[138]
Naphth-AlkyneOMe entrapped in a cross-linked polyvinyl alcohol–glutaraldehyde matrix	10.5–12.5	[139]
